# The protective effect of helmet use in motorcycle and bicycle accidents: a propensity score–matched study based on a trauma registry system

**DOI:** 10.1186/s12889-017-4649-1

**Published:** 2017-08-07

**Authors:** Spencer C. H. Kuo, Pao-Jen Kuo, Cheng-Shyuan Rau, Yi-Chun Chen, Hsiao-Yun Hsieh, Ching-Hua Hsieh

**Affiliations:** 1 Department of Plastic and Reconstructive Surgery, Kaohsiung Chang Gung Memorial Hospital and Chang Gung University College of Medicine, No.123, Ta-Pei Road, Niao-Song District, Kaohsiung City, 833 Taiwan; 2 Department of Neurosurgery, Kaohsiung Chang Gung Memorial Hospital and Chang Gung University College of Medicine, Kaohsiung, Taiwan

**Keywords:** Bicyclist, Motorcyclist, Helmet, Head injury, Injury severity score, Mortality, Trauma registry system

## Abstract

**Background:**

Transportation by motorcycle and bicycle has become popular in Taiwan, this study was designed to investigate the protective effect of helmet use during motorcycle and bicycle accidents by using a propensity score–matched study based on trauma registry system data.

**Methods:**

Data of adult patients hospitalized for motorcycle or bicycle accidents between January 1, 2009 and December 31, 2015 were retrieved from the Trauma Registry System. These included 7735 motorcyclists with helmet use, 863 motorcyclists without helmet use, 76 bicyclists with helmet use, and 647 bicyclists without helmet use. The primary outcome measurement was in-hospital mortality. Secondary outcomes were the hospital length of stay (LOS), intensive care unit (ICU) admission rate, and ICU LOS. Normally distributed continuous data were analyzed by the unpaired Student *t*-test, and non-normally distributed data were compared using the Mann–Whitney *U*-test. Two-sided Fisher exact or Pearson chi-square tests were used to compare categorical data. Propensity score matching (1:1 ratio using optimal method with a 0.2 caliper width) was performed using NCSS software, adjusting for the following covariates: sex, age, and comorbidities. Further logistic regression was used to evaluate the effect of helmet use on mortality rates of motorcyclists and bicyclists, respectively.

**Results:**

The mortality rate for motorcyclists with helmet use (1.1%) was significantly lower than for motorcyclists without helmet use (4.2%; odds ratio [OR] 0.2; 95% confidence interval [CI]: 0.17–0.37; *p* < 0.001). Among bicyclists, there was no significant difference in mortality rates between the patients with helmet use (5.3%) and those without helmet use (3.7%; OR 1.4; 95% CI: 0.49–4.27; *p* = 0.524). After propensity-score matching for covariates, including sex, age, and comorbidities, 856 well-balanced pairs of motorcyclists and 76 pairs of bicyclists were identified for outcome comparison, showing that helmet use among motorcyclists was associated with lower mortality rates (OR 0.2; 95% CI: 0.09–0.44; *p* < 0.001). In contrast, helmet use among bicyclists was not associated with a decrease in mortality (OR 1.3; 95% CI: 0.30–5.96; *p* = 0.706). The hospital LOS was also significantly shorter for motorcyclists with helmet use than for those without (9.5 days vs. 12.0 days, respectively, *p* < 0.001) although for bicyclists, helmet use was not associated with hospital LOS. Fewer motorcyclists with helmet use were admitted to the ICU, regardless of the severity of injury; however, no significant difference of ICU admission rates was found between bicyclists with and without helmets.

**Conclusions:**

Motorcycle helmets provide protection to adult motorcyclists involved in traffic accidents and their use is associated with a decrease in mortality rates and the risk of head injuries. However, no such protective effect of helmet use was observed for bicyclists involved in collisions.

**Electronic supplementary material:**

The online version of this article (doi:10.1186/s12889-017-4649-1) contains supplementary material, which is available to authorized users.

## Background

Traveling by motorcycle has been a common part of daily living for years and remains a crucial mode of transportation in Taiwan, due to the country’s suitable climate and high population density [1, 2]. According to a published study in Taiwan, 3323 persons were killed by traffic accidents in 2011, and more than 60 % of whom were motorcycle riders or passengers [2]. Bicycling has also become popular in Taiwan, not only as a means of transportation, but also as a symbol of personal fitness. In the United States, national statistics report that 500,000 people sustain bicycle-related injuries per year, resulting in approximately 800 deaths [3]. It is estimated that for every 2 million trips, 600 injuries will occur and one bicyclist will die in a collision [4]. In Taiwan, a retrospective cohort study revealed that bicyclists had a 1.2-fold higher adjusted odds ratio (AOR) of in-hospital mortality than motorcyclists [5]. Among patients with injury severity score (ISS) ≥25, bicyclists had a 4.4 times increased odds of mortality compared to motorcyclists (95% confidence interval [CI]: 1.95–9.82) [5].

Although the protective effect of motorcycle helmets is already well-established in the literature [6–14], head injury is still regarded as a critical cause of mortality among victims of motorcycle collisions. Head injury is also the main cause of hospitalization of bicycle-related injuries [15]. Data from an estimate in 2000 noted that approximately $8 billion is spent annually in the United States in the care of bicycle crash victims, which is a significant cost from a public health perspective [16]. Head injuries often occur in motorcycle and bicycle traffic accidents and have severe consequences [17, 18], however, the current laws in Taiwan only enforce the use of helmets on motorcyclists, while helmet use remains optional for bicyclists. As a result, helmet use is more common among motorcycle riders than bicycle riders. Furthermore, most bicycle helmets are smaller, thinner, and lighter, than motorcycle helmets, thus they may provide less protection to riders during collisions. Moreover, because of different riding speeds, the impact energy may differ between collisions involving motorcyclists and those involving bicyclists. There is scarce information on the protective effect of helmet use in Taiwan, where motorcycle and bicycle accidents occur on relatively crowded streets [19]. Therefore, in this study we aimed to investigate the protective effect of helmet use during motorcycle and bicycle accidents by using a propensity score–matched study based on trauma registry system data over a seven-year period.

## Methods

**Fig. 1:**
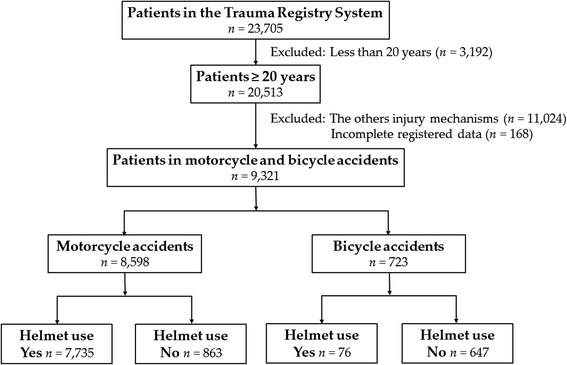
Flow chart of the studied adult trauma population in motorcycle and bicycle accidents

This study was approved by the institutional review board (IRB) of the Kaohsiung Chang Gung Memorial Hospital (reference number 201600005B0), a Level I regional trauma center providing care to trauma patients, primarily from southern Taiwan. We designed a retrospective study to review the data of all adult motorcyclists and bicyclists (*n* = 9321) entered into the Trauma Registry System between January 1, 2009 and December 31, 2015 (Fig. 1). Patients who had sustained an injury other than motorcycle or bicycle accident or whose registered data were incomplete were excluded. Detailed patient information was retrieved from the Trauma Registry System of our institution, including the following variables: age; sex; body mass index (BMI); co-morbidities, such as diabetes mellitus (DM), hypertension (HTN), coronary artery disease (CAD), congestive heart failure (CHF), cerebral vascular accident (CVA), and end-stage renal disease (ESRD); vital signs on arrival; blood alcohol concentration (BAC) on arrival; Glasgow Coma Scale (GCS) score; Abbreviated Injury Scale (AIS); and ISS on arrival; length of stay (LOS) in the hospital and intensive care unit (ICU); in-hospital mortality; procedures performed in the emergency department (ED); and associated head and maxillofacial trauma.

In order to evaluate the relationship between helmet use and traffic accident injury severity and outcome, the data of patients presenting to the ED following motorcycle and bicycle traffic accidents were analyzed. Motorcyclists with helmet use (*n* = 7735) were compared with motorcyclists without helmet use (*n* = 863), and bicyclists with helmet use (*n* = 76) were compared with bicyclists without helmet use (*n* = 647) using the SPSS v.20 statistical software (IBM, Armonk, NY). The primary outcome of the study was in-hospital mortality, and the secondary outcomes were hospital LOS, ICU admission rate, and ICU LOS. For categorical variables, Chi-square tests were used to determine the significance of the associations between the predictor and outcome variables. For continuous variables, Student t-tests were applied to analyze normally distributed continuous data, while Mann-Whitney *U* tests were used to compare non-normally distributed data. Univariate logistic regression analyses were performed to identify the significant predictor variables of the mortality risk. The corresponding crude odds ratio (OR) with 95% CI for each variable was calculated. Pearson’s correlation coefficient (r) was used for bivariate correlation to analyze the relationships between items of co-morbidity, including DM, HTN, CAD, CHF, CVA, and ESRD. Thereafter, to minimize the confounding effects due to a nonrandomized assignment in the evaluation of the effect of helmet use on mortality, propensity scores were calculated using SPSS v.20 statistical software with helmet use as dependent variable and the following covariates as independent variables: sex; age; and comorbidities (Additional file 1: Table S1). Using NCSS software (NCSS 10; NCSS Statistical software, Kaysville, Utah), the optimal method was used to create 1:1 matched study groups with a 0.2 caliper width. After adjusting for these confounding factors, binary logistic regression was used to evaluate the interventional factor of helmet use by motorcyclists and bicyclists on mortality. All results are presented as the mean ± standard deviation. A *p*-value <0.05 was considered statistically significant.

## Results

### Demographics and injury characteristics of patients

The mean age of subjects was 46.0 ± 17.5 years, 48.8 ± 18.6 years, 48.8 ± 13.7 years, and 63.1 ± 15.6 years for motorcyclists with helmet use, motorcyclists without helmet use, bicyclists with helmet use, and bicyclists without helmet use, respectively. The BMI distribution was comparable between motorcyclists with and without helmet use, but in the bicyclist group, significantly more patients had helmets in the overweighted subgroup (35.5% vs. 22.7%, OR: 1.9 [95% CI: 1.13–3.10], *p* = 0.013). Fewer motorcyclists with helmet use had HTN compared to motorcyclists without helmet use. Fewer bicyclists with helmet use had DM and HTN compared with bicyclists without helmet use. Among motorcyclists, significantly fewer patients with helmet use had a BAC level of >50 mg/dl compared to motorcyclists without helmet use (7.1% vs. 24.0%, OR: 0.2 [95% CI: 0.20–0.29], *p* < 0.001). For bicyclists, there was no difference in terms of BAC level between those with helmets and without helmets (2.6% vs. 5.4%, OR: 0.5 [95% CI: 0.11–2.01], *p* = 0.414).

### Covariates and outcome of the patients

The covariates and outcome of adult traffic accident helmet users and nonusers before and after propensity score matching were summarized in Table 1 (motorcyclists) and Table 2 (bicyclists). After propensity score matching, the difference of age, gender, and comorbidities, which included DM, HTN, CAD, CHF, CVA, and ESRD, between the helmet users and non-users was adjusted.

**Table 1:** Covariates and outcome of adult motorcycle traffic accident helmet users and nonusers before and after propensity score matching (1:1 matching)

	Before matching				After matching			
Covariates	Helmet use Yes *n* = 7735	Helmet use No *n* = 863	*Odds ratio* *(95%CI)*	*P*	Helmet use Yes *n* = 856	Helmet use No *n* = 856	Mean Difference	Standardized Difference
Age	46.0 ± 17.5	48.8 ± 18.6	—	<0.001	48.6 ± 18.6	48.6 ± 18.6	−0.01168	−0.06(%)
Sex								
Male	4248(54.9)	592(68.6)	0.6(0.48–0.65)	<0.001	588(68.7)	588(68.7)	1.0(0.82–1.23)	0.00(%)
Female	3487(45.1)	271(31.4)	1.8(1.54–2.09)	<0.001	268(31.3)	268(31.3)	1.0(0.82–1.23)	0.00 (%)
Comorbidity								
DM	831(10.7)	106(12.3)	0.9(0.69–1.07)	0.169	102(11.9)	102(11.9)	1.0(0.75–1.34)	0.00 (%)
HTN	1539(19.9)	201(23.3)	0.8(0.69–0.97)	0.019	197(23.0)	197(23.0)	1.0(0.80–1.25)	0.00 (%)
CAD	156(2.0)	23(2.7)	0.8(0.48–1.17)	0.206	20(2.3)	20(2.3)	1.0(0.53–1.87)	0.00 (%)
CHF	27(0.3)	7(0.8)	0.4(0.19–0.99)	0.076	4(0.5)	4(0.5)	1.0(0.25–4.01)	0.00 (%)
CVA	95(1.2)	15(1.7)	0.7(0.41–1.22)	0.206	11(1.3)	11(1.3)	1.0(0.43–2.32)	0.00 (%)
ESRD	2(0.0)	0(0.0)	—	1.000	0(0.0)	0(0.0)	—	—
Outcome								
GCS	14.4 ± 2.1	12.8 ± 3.8	—	<0.001	14.4 ± 2.0	12.8 ± 3.8	—	<0.001
≤ 8	305(3.9)	145(16.8)	0.2(0.16–0.25)	<0.001	34(4.0)	145(16.9)	0.2(0.14–0.30)	<0.001
9–12	256(3.3)	79(9.2)	0.3(0.26–0.44)	<0.001	31(3.6)	77(9.0)	0.4(0.25–0.58)	<0.001
≥ 13	7174(92.7)	639(74.0)	4.5(3.76–5.34)	<0.001	791(92.4)	634(74.1)	4.3(3.17–5.73)	<0.001
AIS ≥ 3, n (%)								
Head/Neck	1288(16.7)	399(46.2)	0.2(0.20–0.27)	<0.001	149(17.4)	398(46.5)	0.2(0.19–0.30)	<0.001
Face	22(0.3)	7(0.8)	0.3(0.15–0.82)	0.022	4(0.5)	7(0.8)	0.6(0.17–1.95)	0.364
Thorax	685(8.9)	105(12.2)	0.7(0.56–0.87)	0.001	98(11.4)	104(12.1)	0.9(0.70–1.25)	0.653
Abdomen	192(2.5)	21(2.4)	1.0(0.65–1.61)	0.930	21(2.5)	21(2.5)	1.0(0.54–1.85)	1.000
Extremity	1898(24.5)	138(16.0)	1.7(1.41–2.06)	<0.001	190(22.2)	137(16.0)	1.5(1.17–1.91)	0.001
ISS, median (IQR)	8(4–10)	10(5–18)	—	<0.001	9(4–12)	10(5–18)	—	<0.001
< 16	6570(84.9)	530(61.4)	3.5(3.05–4.12)	<0.001	687(80.3)	524(61.2)	2.6(2.07–3.20)	<0.001
16–24	829(10.7)	224(26.0)	0.3(0.29–0.41)	<0.001	144(16.8)	223(26.1)	0.6(0.45–0.73)	<0.001
≥ 25	336(4.3)	109(12.6)	0.3(0.25–0.40)	<0.001	25(2.9)	109(12.7)	0.2(0.13–0.32)	<0.001
Hospital LOS (days)	9.5 ± 9.7	12.0 ± 12.9	—	<0.001	9.5 ± 9.7	12.0 ± 12.9	—	<0.001
ICU								
Patients, n (%)	1291(16.7)	338(39.2)	0.3(0.27–0.36)	<0.001	156(18.2)	336(39.3)	0.3(0.28–0.43)	<0.001
< 16	447(5.8)	85(9.8)	0.6(0.44–0.72)	<0.001	48(5.6)	84(9.8)	0.5(0.38–0.80)	0.001
16–24	545(7.0)	155(18.0)	0.3(0.29–0.42)	<0.001	83(9.7)	154(18.0)	0.5(0.37–0.65)	<0.001
≥ 25	299(3.9)	98(11.4)	0.3(0.25–0.40)	<0.001	25(2.9)	98(11.4)	0.2(0.15–0.37)	<0.001
ICU LOS (days)	6.9 ± 8.3	7.6 ± 9.0	—	0.172	6.1 ± 6.8	7.6 ± 9.1	—	0.043
Mortality	83(1.1)	36(4.2)	0.2(0.17–0.37)	<0.001	7(0.8)	36(4.2)	0.2(0.09–0.44)	<0.001

*CI* confidence interval, *BMI* body mass index, *DM* diabetes mellitus, *HTN* hypertension, *CAD* coronary artery disease, *CHF* congestive heart failure, *CVA* cerebral vascular accident, *ESRD* end-stage renal disease, *BAC* blood alcohol concentration, *GCS* Glasgow Coma Scale, *AIS* Abbreviated Injury Scale, *ISS* injury severity score, *IQR interquartile range*, *LOS* length of stay, *ICU* intensive care unit

**Table 2:** Covariates and outcome of adult bicycle traffic accident helmet users and nonusers before and after propensity score matching (1:1 matching)

	Before matching				After matching			
Covariates	Helmet use Yes *n* = 76	Helmet use No *n* = 647	*Odds ratio* *(95%CI)*	*P*	Helmet use Yes *n* = 76	Helmet use No *n* = 76	Mean Difference	Standardized Difference
Age	48.8 ± 13.7	63.1 ± 15.6	—	<0.001	48.8 ± 13.7	48.7 ± 13.8	0.09211	0.67(%)
Sex								
Male	60(78.9)	355(54.9)	3.1(1.74–5.47)	<0.001	60(78.9)	60(78.9)	1.0(0.46–2.18)	0.00(%)
Female	16(21.1)	292(45.1)	0.3(0.18–0.58)	<0.001	16(21.1)	16(21.1)	1.0(0.46–2.18)	0.00 (%)
Comorbidity								
DM	3(3.9)	86(13.3)	0.3(0.08–0.87)	0.019	3(3.9)	3(3.9)	1.0(0.20–5.12)	0.00 (%)
HTN	12(15.8)	221(34.2)	0.4(0.19–0.68)	0.001	12(15.8)	12(15.8)	1.0(0.42–2.39)	0.00 (%)
CAD	1(1.3)	22(3.4)	0.4(0.05–2.85)	0.498	1(1.3)	1(1.3)	1.0(0.06–16.29)	0.00 (%)
CHF	0(0.0)	4(0.6)	—	1.000	0(0.0)	0(0.0)	—	—
CVA	0(0.0)	28(4.3)	—	0.063	0(0.0)	0(0.0)	—	—
ESRD	0(0.0)	2(0.3)	—	1.000	0(0.0)	0(0.0)	—	—
Outcome								
GCS	14.5 ± 2.4	14.2 ± 2.4	—	0.303	14.5 ± 2.4	14.2 ± 2.6	—	0.538
≤ 8	3(3.9)	34(5.3)	0.7(0.22–2.47)	0.788	3(3.9)	4(5.3)	0.7(0.16–3.42)	1.000
9–12	0(0.0)	31(4.8)	—	0.065	0(0.0)	2(2.6)	—	0.497
≥ 13	73(96.1)	582(90.0)	2.7(0.83–8.87)	0.085	73(96.1)	70(92.1)	2.1(0.50–8.67)	0.494
AIS ≥ 3, n (%)								
Head/Neck	9(11.8)	166(25.7)	0.4(0.19–0.80)	0.008	9(11.8)	14(18.4)	0.6(0.24–1.47)	0.258
Face	0(0.0)	1(0.2)	—	1.000	0(0.0)	0(0.0)	—	—
Thorax	4(5.3)	33(5.1)	1.0(0.36–3.00)	1.000	4(5.3)	3(3.9)	1.4(0.29–6.26)	1.000
Abdomen	0(0.0)	7(1.1)	—	1.000	0(0.0)	0(0.0)	—	—
Extremity	20(26.3)	208(32.1)	0.8(0.44–1.29)	0.301	20(26.3)	19(25.0)	1.1(0.52–2.22)	0.853
ISS, median (IQR)	5.5(4–9)	9(4–10)	—	0.029	5.5(4–9)	7(4–10)	—	0.808
< 16	68(89.5)	523(80.8)	2.0(0.94–4.30)	0.065	68(89.5)	63(82.9)	1.8(0.68–4.51)	0.240
16–24	5(6.6)	88(13.6)	0.4(0.18–1.14)	0.084	5(6.6)	11(14.5)	0.4(0.14–1.26)	0.113
≥ 25	3(3.9)	36(5.6)	0.7(0.21–2.32)	0.788	3(3.9)	2(2.6)	1.5(0.25–9.37)	1.000
Hospital LOS (days)	7.0 ± 9.5	8.9 ± 10.2	—	0.116	7.0 ± 9.5	6.7 ± 6.4	—	0.858
ICU								
Patients, n (%)	9(11.8)	139(21.5)	0.5(0.24–1.01)	0.049	9(11.8)	11(14.5)	0.8(0.31–2.04)	0.631
< 16	4(5.3)	38(5.9)	0.9(0.31–2.57)	1.000	4(5.3)	1(1.3)	4.2(0.46–38.17)	0.367
16–24	2(2.6)	69(10.7)	0.2(0.05–0.94)	0.026	2(2.6)	8(1.1)	0.2(0.05–1.12)	0.050
≥ 25	3(3.9)	32(4.9)	0.8(0.24–2.64)	1.000	3(3.9)	2(2.6)	1.5(0.25–9.37)	1.000
ICU LOS (days)	11.0 ± 20.1	7.4 ± 9.5	—	0.612	11.0 ± 20.1	4.3 ± 2.3	—	0.346
Mortality	4(5.3)	24(3.7)	1.4(0.49–4.27)	0.524	4(5.3)	3(3.9)	1.3(0.30–5.96)	0.706

*CI* confidence interval, *BMI* body mass index, *DM* diabetes mellitus, *HTN* hypertension, *CAD* coronary artery disease, *CHF* congestive heart failure, *CVA* cerebral vascular accident, *ESRD* end-stage renal disease, *BAC* blood alcohol concentration, *GCS* Glasgow Coma Scale, *AIS* Abbreviated Injury Scale, *ISS* injury severity score, *IQR interquartile range*, *LOS* length of stay, *ICU* intensive care unit

For the motorcyclist group before matching (Table 1), the average GCS score was significantly higher for patients with helmet use. Significantly fewer motorcyclists with helmet use had a GCS score ≤ 12 upon arrival to the ED and a significantly greater number had a GCS score ≥ 13 compared with motorcyclists without helmet use. Fewer patients with helmet use had an AIS ≥3 over the head/neck, face, thorax, and extremities, compared with patients without helmet use. A lower median ISS (8 [4–10] vs. 10 [5–18], *p* < 0.001) was also observed in motorcyclists with helmet use and significantly fewer patients were severely injured (ISS between 16 and 24 and ≥25) compared with patients without helmet use. The hospital LOS was also significantly shorter for motorcyclists with helmet use than for those without helmet use (9.5 days vs. 12.0 days, respectively, *p* < 0.001), and significantly fewer patients with helmet use were admitted to the ICU. No significant difference of the ICU LOS was found. The mortality rate for motorcyclists with helmet use was significantly lower than for motorcyclists without helmet use (1.1% vs. 4.2%, OR: 0.2 [95% CI: 0.17–0.37], *p* < 0.001). After propensity score matching, the outcomes were generally comparable with those before matching. However, the ICU LOS came out significantly shorter for motorcyclists with helmet use (6.1 ± 6.8 days vs. 7.6 ± 9.1 days, *p* = 0.043). The mortality remained significantly lower for motorcyclists with helmet use (0.8% vs. 4.2%, OR: 0.2 [95% CI: 0.09–0.44], *p* < 0.001).

For the bicyclist group before propensity score matching (Table 2), no significant difference in terms of GCS was found between those with and without helmet use. Significantly fewer patients with helmet use had an AIS ≥3 over the head/neck region. The median ISS was also significantly lower (5.5 [4–9] vs. 9 [4–10], *p* = 0.029) for bicyclists with helmet use than for those without helmet use. However, the distribution of ISS according to the stratification of ISS (<16, 16–24, ≥25) showed no significant difference between the helmet users and non-users. The hospital LOS and ICU LOS were not significantly different between bicyclists with or without helmet use, but fewer bicyclists with helmet use were admitted to the ICU. There was no significant difference in mortality rates among bicyclists, with or without helmet use (5.3% vs. 3.7%, OR: 1.4 [95% CI: 0.49–4.27], *p* = 0.524). After propensity score matching, there was no significant difference in terms of the GCS, AIS ≥ 3, and the ISS among bicyclists with or without helmet use. The hospital LOS and ICU LOS were also comparable between the two groups. Still, helmet use in bicyclists was not significantly associated with mortality in this study, although the odds of mortality in bicyclists with helmet use was 1.3 times that of bicyclists without helmet use (OR: 1.3 [95% CI: 0.30–5.96], *p* = 0.706).

### Associated injuries at ED

Physiological parameters and life-saving procedures, including cardiopulmonary resuscitation, intubation, chest tube insertion, and blood transfusion performed in the ED are summarized in Table 3 (motorcyclists) and Table 4 (bicyclists). In the motorcyclists group, helmet use was associated with a decreased number of patients with a heart rate > 100 beat/min and a respiratory rate < 10/min or >29/min, and significantly lower intubation rates. After propensity score matching, we only found a significantly lower intubation rate for motorcyclists with helmet use. For bicyclists, helmet use was not associated with a significantly improved physiological response or with fewer life-saving procedures before or after propensity score matching.

**Table 3:** Physiological parameters and life-saving procedures performed at ED for adult motorcycle traffic accident trauma patients

	Original cohort				Propensity score-matched cohort			
Parameters	Helmet use Yes *n* = 7735	Helmet use No *n* = 863	*Odds ratio* *(95%)*	*P*	Helmet use Yes *n* = 856	Helmet use No *n* = 856	*Odds ratio* *(95%)*	*P*
Physiology at ED, n (%)								
SBP < 90 mmHg	163(2.1)	24(2.8)	0.8(0.49–1.16)	0.198	18(2.1)	24(2.8)	0.7(0.40–1.38)	0.349
Heart rate > 100 beats/min	1363(17.6)	182(21.1)	0.8(0.67–0.95)	0.012	159(18.6)	181(21.1)	0.9(0.67–1.08)	0.183
Respiratory rate < 10 or >29 /min	34(0.4)	11(1.3)	0.3(0.17–0.68)	0.004	5(0.6)	11(1.3)	0.5(0.16–1.31)	0.132
Procedures at ED, n (%)								
Cardiopulmonary resuscitation	8(0.1)	2(0.2)	0.4(0.09–2.10)	0.265	1(0.1)	2(0.2)	0.5(0.05–5.52)	1.000
Intubation	147(1.9)	57(6.6)	0.3(0.20–0.38)	<0.001	17(2.0)	57(6.7)	0.3(0.16–0.49)	<0.001
Chest tube insertion	108(1.4)	16(1.9)	0.8(0.44–1.27)	0.285	14(1.6)	16(1.9)	0.9(0.42–1.80)	0.713
Blood transfusion	266(3.4)	38(4.4)	0.8(0.55–1.09)	0.146	37(4.3)	38(4.4)	1.0(0.61–1.55)	0.906

ED = emergency department; SBP = systolic blood pressure

**Table 4:** Physiological parameters and life-saving procedures performed at ED for adult bicycle traffic accident trauma patients

	Original cohort				Propensity score-matched cohort			
Parameters	Helmet use Yes *n* = 76	Helmet use No *n* = 647	*Odds ratio* *(95%)*	*P*	Helmet use Yes *n* = 76	Helmet use No *n* = 76	*Odds ratio* *(95%)*	*P*
Physiology at ED, n (%)								
SBP < 90 mmHg	1(1.3)	13(2.0)	0.7(0.08–5.04)	1.000	1(1.3)	1(1.3)	1.0(0.06–16.29)	1.000
Heart rate > 100 beats/min	7(9.2)	105(16.2)	0.5(0.23–1.17)	0.110	7(9.2)	9(11.8)	0.8(0.27–2.14)	0.597
Respiratory rate < 10 or >29 /min	0(0.0)	5(0.8)	—	1.000	0(0.0)	0(0.0)	—	—
Procedures at ED, n (%)								
Cardiopulmonary resuscitation	1(1.3)	2(0.3)	4.3(0.39–47.99)	0.284	1(1.3)	0(0.0)	—	1.000
Intubation	1(1.3)	19(2.9)	0.4(0.06–3.34)	0.712	1(1.3)	1(1.3)	1.0(0.06–16.29)	1.000
Chest tube insertion	0(0.0)	7(1.1)	—	1.000	0(0.0)	1(1.3)	—	1.000
Blood transfusion	2(2.6)	23(3.6)	0.7(0.17–3.17)	1.000	2(2.6)	1(1.3)	2.0(0.18–22.84)	1.000

*ED* emergency department, *SBP* systolic blood pressure

Regarding the associated injuries in the head and face region, significantly fewer motorcyclists with helmet use suffered head and maxillofacial trauma, including neurological deficit, cranial fracture, epidural hematoma (EDH), subdural hematoma (SDH), subarachnoid hemorrhage (SAH), intracerebral hemorrhage (ICH), cerebral contusion, cervical vertebra fracture, nasal fracture, maxillary fracture, and mandibular fracture were found than those motorcyclists without helmet use before propensity score matching (Table 5). However, this protective effect of helmets was not observed in the bicyclists group, except there was a reduced risk of developing SDH among bicyclists with helmet use than those without (2.6% vs. 13.2%, OR: 0.2 [95% CI: 0.04–0.84], *p* = 0.031) (Table 6). After propensity score matching, the same protective effect of helmets could still be observed in the motorcycle group, but not in the bicycle group.

**Table 5:** Associated injuries among adult motorcycle traffic accident trauma patients

		Original cohort				Propensity score-matched cohort		
Associated injuries	Helmet use Yes *n* = 7735	Helmet use No *n* = 863	*Odds ratio* *(95%)*	*P*	Helmet use Yes *n* = 856	Helmet use No *n* = 856	*Odds ratio* *(95%)*	*P*
Head trauma, n (%)								
Neurologic deficit	69(0.9)	15(1.7)	0.5(0.29–0.89)	0.017	8(0.9)	15(1.8)	0.5(0.22–1.25)	0.142
Cranial fracture	444(5.7)	175(20.3)	0.2(0.20–0.29)	<0.001	53(6.2)	173(20.2)	0.3(0.19–0.36)	<0.001
Epidural hematoma (EDH)	283(3.7)	124(14.4)	0.2(0.18–0.28)	<0.001	38(4.4)	12(14.5)	0.3(0.19–0.40)	<0.001
Subdural hematoma (SDH)	647(8.4)	245(28.4)	0.2(0.20–0.27)	<0.001	87(10.2)	245(28.6)	0.3(0.22–0.37)	<0.001
Subarachnoid hemorrhage (SAH)	770(10.0)	228(26.4)	0.3(0.26–0.36)	<0.001	91(10.6)	227(26.5)	0.3(0.25–0.43)	<0.001
Intracerebral hemorrhage (ICH)	169(2.2)	48(5.6)	0.4(0.27–0.53)	<0.001	19(2.2)	48(5.6)	0.4(0.22–0.66)	<0.001
Cerebral contusion	353(4.6)	120(13.9)	0.3(0.24–0.37)	<0.001	43(5.0)	120(14.0)	0.3(0.23–0.47)	<0.001
Cervical vertebra fracture	60(0.8)	15(1.7)	0.4(0.25–0.78)	0.004	4(0.5)	15(1.8)	0.3(0.09–0.80)	0.011
Maxillofacial trauma, n (%)								
Nasal fracture	111(1.4)	20(2.3)	0.6(0.38–0.99)	0.045	12(1.4)	20(2.3)	0.6(0.29–1.22)	0.153
Maxillary fracture	731(9.5)	123(14.3)	0.6(0.51–0.77)	<0.001	85(9.9)	122(14.3)	0.7(0.49–0.89)	0.006
Mandibular fracture	234(3.0)	39(4.5)	0.7(0.47–0.93)	0.018	21(2.5)	39(4.6)	0.5(0.31–0.90)	0.018

**Table 6:** Associated injuries among adult bicycle traffic accident trauma patients

		Original cohort				Propensity score-matched cohort		
Associated injuries	Helmet use Yes *n* = 76	Helmet use No *n* = 647	*Odds ratio* *(95%)*	*P*	Helmet use Yes *n* = 76	Helmet use No *n* = 76	*Odds ratio* *(95%)*	*P*
Head trauma, n (%)								
Neurologic deficit	0(0.0)	3(0.5)	—	1.000	0(0.0)	0(0.0)	—	—
Cranial fracture	4(5.3)	37(5.7)	0.9(0.32–2.64)	1.000	4(5.3)	5(6.6)	0.8(0.20–3.06)	1.000
Epidural hematoma (EDH)	1(1.3)	34(5.3)	0.2(0.03–1.78)	0.163	1(1.3)	5(6.6)	0.2(0.02–1.66)	0.209
Subdural hematoma (SDH)	2(2.6)	95(14.7)	0.2(0.04–0.65)	0.004	2(2.6)	10(13.2)	0.2(0.04–0.84)	0.031
Subarachnoid hemorrhage (SAH)	8(10.5)	80(12.4)	0.8(0.39–1.80)	0.643	8(10.5)	9(11.8)	0.9(0.32–2.41)	0.797
Intracerebral hemorrhage (ICH)	1(1.3)	16(2.5)	0.5(0.07–4.02)	1.000	1(1.3)	0(0.0)	—	1.000
Cerebral contusion	3(3.9)	45(7.0)	0.6(0.17–1.81)	0.319	3(3.9)	4(5.3)	0.7(0.16–3.42)	1.000
Cervical vertebra fracture	2(2.6)	9(1.4)	1.9(0.41–9.04)	0.325	2(2.6)	1(1.3)	2.0(0.18–22.84)	1.000
Maxillofacial trauma, n (%)								
Nasal fracture	1(1.3)	5(0.8)	1.7(0.20–14.85)	0.488	1(1.3)	2(2.6)	0.5(0.04–5.56)	1.000
Maxillary fracture	9(11.8)	34(5.3)	2.4(1.11–5.27)	0.035	9(11.8)	4(5.3)	2.4(0.71–8.22)	0.147
Mandibular fracture	2(2.6)	9(1.4)	1.9(0.41–9.04)	0.325	2(2.6)	0(0.0)	—	0.497

## Discussion

The results of this study highlight the protective effect of motorcycle helmets, with a significantly lower mortality rate among motorcyclists with helmet use than among motorcyclists without helmet use. The well-balanced propensity score-matched model, which eliminated the confounding effects of sex, age, and comorbidities, further strengthened our conclusion on the protective effect of motorcycle helmets. Furthermore, motorcycle helmet users also had higher GCS scores and a lower ISS scores on presentation, shorter hospital LOS, and lower ICU admission rates. The protective effect of bicycle helmets was not demonstrated in the bicyclists group, despite the use of propensity score-matched populations of bicyclists.

The protective effect of motorcycle helmets is well established in the literature [6–14]. Helmet use among motorcyclists was found to benefit both riders and society, with improved discharge outcomes, as well as a reduction in mortality rates, traumatic brain injuries, and costs of hospitalization [6–14]. Hooten et al. concluded that motorcycle helmets significantly decrease overall mortality, improve outcome at discharge, and are cost-effective, resulting in healthcare savings [6]. A systemic review published in 2008 by Liu et al. also concluded that motorcycle helmets reduce the risk of death and head injury among motorcycle riders involved in collisions [7]. Another retrospective study conducted by Sosin et al., found that motorcycle helmets not only reduce the severity of nonfatal head injuries, but also lower the rate of fatal injuries [8]. The legal enforcement of helmet use for motorcycle riders is therefore of crucial importance. Kraus et al. found that the enactment of motorcycle helmet law in California of USA significantly reduces the fatality rate in motorcycle accidents, as well as the number and severity of head injuries [9]. Moreover, Hotz et al. reported a significantly increased number and severity of brain injuries following the repeal of a motorcycle helmet law [10]. The data on cervical spine protection is controversial, with some studies reporting that the use of motorcycle helmets may increase the rate of cervical spine injury [20, 21], and others reporting statistically similar, or even lower risks of cervical spine injury [22–26]. In this study, although the neurological damage from cervical spine injury was not evaluated, motorcycle helmet use was associated with a significantly lower rate of cervical vertebra fractures.

The protective effect of bicycle helmets has been reported in the literature. Heng et al. found that helmet use was associated with fewer injuries to the head and face, as well as a lower ISS, in bicycle crashes [15]. In two case-control studies published by Thompson et al., bicycle helmet use was associated with a decreased risk of head injuries among injured bicyclists [27, 28]. A case-control study also reported a 74% reduced risk of head injury among bicyclists with helmet use involved in accidents with motor vehicles [29]. Another recent study of 13,500 bicyclist injuries in France, conducted by Amoros et al., found a lower risk of all head injuries among bicyclists with helmet use, in both urban and rural environments [30]. However, Sethi et al. studied injured bicyclists in a level I regional trauma center and found no significant difference in mortality rates between patients with and those without helmet use [3]. In this study, we only observed a reduced risk of developing SDH among bicyclists with helmet use compared to those without after propensity score matching, while the mortality rate and the risks of all the other associated head injuries, other than SDH, were not significantly reduced. Therefore, unlike helmet use among motorcyclists, protective effect of helmet use in lowering mortality rates could not be identified among bicyclists.

A number of reasons for the lack of a protective effect of bicycle helmets in lowering mortality rates and rates of a range of head injuries in this study were identified. Firstly, the design and material of bicycle helmets are significantly different to those of motorcycle helmets, thus less protection could be expected. Secondly, it is reasonable to postulate that the distinct contusion force and trauma mechanism of bicycle traffic accidents can result in differences in the severity and presentation of intracranial hemorrhage between traffic accidents involving bicyclists and those involving motorcyclists. Therefore, the protective effect of bicycle helmets against death and different types of head injuries could not be demonstrated in relatively minor crashes. Thirdly, the sample size of bicyclists in our study may not be sufficiently large to generate a significant difference in mortality rates and rates of head injuries between bicyclists with and without helmet use. Further studies involving a larger series or involving a controlled crash scenario may provide more robust evidence of a protective effect of bicycle helmets.

A specific strength of our study was the propensity score-matching model, which markedly reduced bias on mortality. However, the results after matching depended on the specification of the logistic regression model and the potential confounders measured in this study. Therefore, balance between the comparison groups regarding unmeasured confounders could not be fully guaranteed. This study also presented a number of limitations. First, the inherent bias of retrospective studies must be considered. Second, the lack of available data regarding the circumstances of the crash, the mechanism of injury, the number of riders and their status of helmet-wearing on the same motorcycle or bike, the speed of the motorcycle or bike, and the type of helmet used, could also lead to a bias. Third, injured patients who were discharged against advice from the ED, those who were not brought to a level I trauma center after the crash, on-site deaths, those who were discharged but got re-admitted into hospital not via the ED, and those who were discharged but died from a complication directly related to the trauma, were not included in this study. This may represent a sample bias in the assessment of mortality. Notably, because there were more on-site deaths in motorcycle crashes than in bicycle crashes in our hospital, the extent of difference in terms of mortality in this study would be expected to be greater in the motorcyclists compared with that in the bicyclists. In addition, other important data, such as surgical outcome and complications, were not evaluated in this study. Finally, the study population, limited to a single urban trauma center in southern Taiwan, may not be representative of other populations.

## Conclusion

This study, conducted at a Level I trauma center, revealed that motorcycle helmets provide protection to adult motorcyclists involved in traffic accidents and their use is associated with a decrease in mortality rates and the risk of different head injuries. Motorcycle helmet users also had a higher GCS score and a lower ISS score, a shorter hospital LOS, and a lower ICU admission rate. In contrast, for bicyclists, helmet use was not associated with a decrease in mortality rates and the risk of different head injuries other than SDH. This study therefore did not identify any protective effect of bicycle helmets for adult bicyclists involved in traffic accidents in Taiwan to keep them away from the risk of different head injuries and mortality.

## Additional file

Additional file 1: Table S1. Bivariate correlation among co-morbidities of the patients in motorcycle and bicycle accidents and the calculated propensity scores according to age, sex, and comorbidities. (DOCX 16 kb)
