# Supplementary material for: The protective effect of helmet use in motorcycle and bicycle accidents: a propensity score–matched study based on a trauma registry system
Source: BMC Public Health. 2017 Aug 7;17:639. doi: 10.1186/s12889-017-4649-1 (PMC5545860; doi:10.1186/s12889-017-4649-1)
Supplement: Additional file 1: Table S1. — Bivariate correlation among co-morbidities of the patients in motorcycle and bicycle accidents and the calculated propensity scores according to age, sex, and comorbidities. (DOCX 16 kb) [file 12889_2017_4649_MOESM1_ESM.docx]

**Motorcycle accident**

**Variables comparison with contingency coefficient (r)**

| **Variables** | **DM** | **HTN** | **CAD** | **CHF** | **CVA** | **ESRD** |
| --- | --- | --- | --- | --- | --- | --- |
| **DM** |  | 0.346 | 0.161 | 0.055 | 0.102 | 0.005 |
| **HTN** |  |  | 0.202 | 0.051 | 0.157 | 0.011 |
| **CAD** |  |  |  | 0.069 | 0.070 | 0.002 |
| **CHF** |  |  |  |  | 0.026 | 0.001 |
| **CVA** |  |  |  |  |  | 0.002 |
| **ERSD** |  |  |  |  |  |  |

**Propensity score calculated by logistic regression (SPSS)**

Log (P_helmet use=1_/1-P_helmet use=1_)=3.081-0.629*(Sex=1)-0.010*Age+0.013*(DM=1)-0.031*(HTN=1)+0.023*(CAD=1)-0.627*(CHF=1)-0.045*(CVA=1)+18.558*(ESRD=1)

**Bicycle accident**

**Variables comparison with contingency coefficient (r)**

| **Variables** | **DM** | **HTN** | **CAD** | **CHF** | **CVA** | **ESRD** |
| --- | --- | --- | --- | --- | --- | --- |
| **DM** |  | 0.247 | 0.146 | 0.029 | 0.056 | 0.020 |
| **HTN** |  |  | 0.160 | 0.012 | 0.195 | 0.020 |
| **CAD** |  |  |  | 0.014 | 0.004 | 0.010 |
| **CHF** |  |  |  |  | 0.015 | 0.004 |
| **CVA** |  |  |  |  |  | 0.125 |
| **ERSD** |  |  |  |  |  |  |

**Propensity score calculated by logistic regression(SPSS)**

Log (P_helmet use=1_/1-P_helmet use=1_)=-0.146+0.844*(Sex=1)-0.043*Age-0.781*(DM=1)-0.128*(HTN=1)-0.030*(CAD=1)-18.964*(CHF=1)-18.495*(CVA=1)-16.780*(ESRD=1)
